# Sex Differences in Brain Transcriptomes of Juvenile Cynomolgus Macaques

**DOI:** 10.3390/biom15050671

**Published:** 2025-05-06

**Authors:** Nadia Kabbej, Frederick J. Ashby, Alberto Riva, Paul D. Gamlin, Ronald J. Mandel, Aishwarya Kunta, Courtney J. Rouse, Coy D. Heldermon

**Affiliations:** 1 Department of Medicine, College of Medicine, University of Florida, Gainesville, FL 32610, USA; nkabbej@ufl.edu (N.K.); ricky.ashby@ufl.edu (F.J.A.); kuntaa@acom.edu (A.K.); cjrouse@ufl.edu (C.J.R.); 2 Bioinformatics Core, Interdisciplinary Center for Biotechnology Research, University of Florida, Gainesville, FL 32608, USA; 3 Department of Ophthalmology & Visual Sciences, University of Alabama at Birmingham, Birmingham, AL 35294, USA; pgamlin@uab.edu; 4 Department of Neuroscience, University of Florida, Gainesville, FL 32610, USA; rmandel@ufl.edu

**Keywords:** sexual dimorphism, pre-pubertal, cynomolgus macaques, transcriptomics, translation, COVID-19, behavior, immunity

## Abstract

Background: Behavioral, social, and physical characteristics are posited to distinguish the sexes, yet research on transcription-level sexual differences in the brain is limited. Here, we investigated sexually divergent brain transcriptomics in pre-pubertal cynomolgus macaques, a commonly used surrogate species to humans. Methods: A transcriptomic profile using RNA sequencing was generated for the temporal lobe, ventral midbrain, and cerebellum of three female and three male cynomolgus macaques previously treated with an adeno-associated virus vector mix. Statistical analyses to determine differentially expressed protein-coding genes in all three lobes were conducted using DeSeq2 with a false-discovery-rate-corrected *p*-value of 0.05. Results: We identified target genes in the temporal lobe, ventral midbrain, and cerebellum with functions in translation, immunity, behavior, and neurological disorders that exhibited statistically significant sexually divergent expression. Conclusions: We provide potential mechanistic insights into the epidemiological differences observed between the sexes with regard to mental health and infectious diseases, such as COVID-19. Our results provide pre-pubertal information on sexual differences in non-human primate brain transcriptomics and may provide insight into health disparities between the biological sexes in humans.

## 1. Introduction

Non-human primates (NHPs) are the closest phylogenetic surrogate model used in research to emulate the human brain [1]. Macaque models are critical to neurophysiology research because they share a significant overlap in structure and function with humans. Diverging from humans an estimated 26.8 million years ago, macaques share >93% gene-sequence similarity to that of humans [2,3]. Neocortical size is also comparable, consisting of 72% of the brain volume in rhesus macaques and 80% in humans. Humans and macaques were also found to share structural homology in the rich club structures of their connectome, suggesting the route of information processing may be similar [4]. These similarities in structure and higher-order brain function make macaques a robust and preferred model for human neuroscientific research.

Anatomical sexual dimorphism in the human brain has been described with relatively new neuroimaging techniques such as Magnetic Resonance Imaging (MRI). In humans, studies have found differences in cerebral volumes, white-matter microstructure, neural connectivity, emotional processing, and visuospatial abilities [5,6]. The literature specific to NHPs is limited concerning sexual dimorphism and its characteristics. Studies using structural MRI in rhesus macaques (MM) reported a larger overall brain volume and corpus callosum in males, and a larger striatum and hippocampus in females [7,8]. A later study in Capuchin monkeys reported an enlarged hypothalamus in males and a larger cerebellum and visual cortex in females [9]. These conflicting findings demonstrate a need for more in-depth research methods beyond anatomical differences to ascertain a complete understanding of sexual differences in macaques. Further, there is little research on NHP sexual differences in brain transcriptomics [10]. Current research on the transcriptomics of macaques has revealed similarities to human expression profiles. Yin et al. compared the transcriptomic profile of a human brain with 52 brain regions in rhesus macaques and reported that humans and macaques have similar expression profiles in the thalamus, striatum, and cerebellar cortex, ref. [11], suggesting them as a valid model for transcriptomic comparisons. Another study by DeCasien et al. reinforced these findings, comparing sex differences in gene expression profiles between adult rhesus macaques and humans across 15 brain regions. Results showed that 51.9% of one-to-one orthologous genes demonstrated conserved sex-specific expression in at least one brain region between humans and macaques [12]. Tissue-specific microglial transcriptome in rhesus macaques has also been explored, where genes exhibited comparable expression levels to humans [13]. Macaque transcriptomic profiles, therefore, show reasonable translatability to humans and should provide important baseline data for researchers to use in future studies.

**Figure 1:**
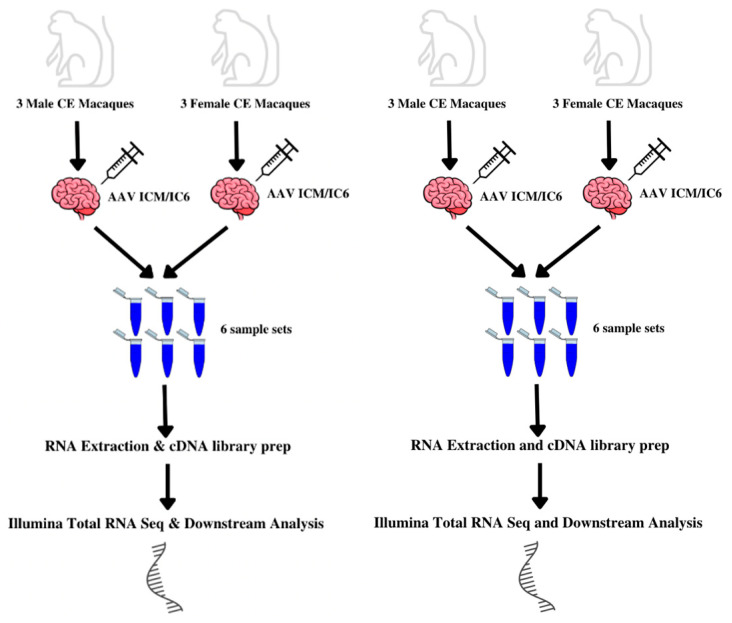
Experimental design.

In this study, we determine the degree of sex differences present in the transcriptomics of CE macaques. We carry out transcriptomic profiling on six young, pre-pubertal CE macaques previously treated with a barcoded adeno-associated virus (AAV) vector mix, using RNA-seq on samples obtained from the ventral midbrain, cerebellum, and temporal lobe (Figure 1). We then conduct a male-to-female comparison of the expression levels of genes found in each brain region followed by a functional annotation analysis of processes involving these genes.

## 2. Materials and Methods

### 2.1. Animal Care, Brain Dissection, and Sample Processing

Six cynomolgus macaques—three males and three females, aged between 28 and 32 months—were procured from Bioculture Group (Glenmoor, PA, USA), then housed and cared for at the University of Alabama in Birmingham. In compliance with USDA’s Animal Welfare Act and Animal Welfare Regulations, the study methods for this project strictly adhered to the recommendations located in the *Guide for the Care and Use of Laboratory Animals*, as outlined by the National Institutes of Health. The Institutional Animal Care and Use Committee (IACUC-Protocol 21022, approved 2017) at the University of Alabama in Birmingham oversaw and approved all protocols and all animals were maintained in an AAALAC-accredited facility. Brain sample collection and animal euthanasia followed the standardized protocol previously described by Kondratov et al. [14]. Immediately following euthanasia, the temporal lobe, ventral midbrain, and cerebellum were dissected by one trained neuroscientist knowledgeable on CE macaque anatomy, Dr. Ron Mandel, in the Department of Neuroscience at the University of Florida. For a more detailed description of dissection landmarks, see Kondratov et al. [14]. Dual punch biopsies (one for DNA and RNA extraction) for the temporal lobe, ventral midbrain, and cerebellum were then placed in a phosphate buffer and frozen immediately in liquid nitrogen prior to RNA extraction.

### 2.2. RNA Extraction and cDNA Library Preparation

RNA isolation and purification were carried out by the Toxicology Core Facility at the University of Florida. For the temporal lobe, ventral midbrain, and cerebellum, RNA was isolated and prepared for extraction as previously described by Kondratov et al. [14]. Total RNA for each sample was then extracted using a QIAGEN RNeasy^®^ Lipid Tissue kit (Cat #74804) according to the manufacturer’s instructions. Each sample’s RNA Integrity Number (RIN) was determined using the Agilent 2100 Bioanalyzer to ensure the RIN for each sample was greater than seven.

Post-RNA extraction, a quality-controlled cDNA library was prepared and carried out by the University of Florida Interdisciplinary Center for Biotechnology Research (ICBR) in the NextGen DNA Sequencing (NGS) core facility. Synthesis of cDNA libraries required 50 ng of RNA per sample, and cDNA synthesis reactions were performed using the NEBNext^®^ Strand-switch cDNA synthesis reagents (Cat #E6421). Reactions were first optimized using the RNA samples with the lowest RIN. Amplification reactions were performed using NEBNext^®^ High-Fidelity 2X PCR Master Mix (Cat# M0541) with the following conditions: denaturation at 98 °C for 45 s, then cycling at 98 °C for 10 s, 62 °C for 15 s, 72 °C for 3 min, and a final extension at 65 °C for 5 min. Each cDNA product underwent quality control using the Invitrogen Qubit^®^ 3.0. Quality control was further ensured by testing every fourth sample using the Agilent 2200 Tape Station; at least 5 Tape Station runs were completed. Libraries were amplified for 14 rounds, then sequenced on the Illumina NovaSeq6000. Overall, this generated 1,418,598,125 total reads with a percent quality score greater than 30. These same optimized conditions were used to synthesize and amplify double-stranded cDNA for all samples.

### 2.3. RNAseq Processing, Quality Control, and Normalization

Fastq files generated from the Illumina workflow underwent read-level quality control on original and trimmed reads using FAST QC (v0.11.4) and MultiQC [15]. Read lengths were all 2 × 150 paired-end reads. Short reads were trimmed using Trimmomatic (v0.36) [16]; about 95% of the reads were retained after trimming. The reads were then aligned to the reference transcriptomes (ENSEMBL Macaca_fascicularis_6.0 and ENSEMBL Macaca_Mulatta_8.0) using STAR (v2.7.9a) [17], and transcript abundance was quantified using RSEM (RSEM v1.3.1) [18]. Finally, differential expression analysis was performed using DESeq2 [19] with an FDR-corrected *p*-value of 0.05. The output files were then filtered to extract transcripts showing a 2.0-fold change in either direction.

### 2.4. Gene Ontology Enrichment Analysis and Pathway Analysis

Previously identified significant gene sets from bulk RNAseq analysis in the temporal lobe, cerebellum, and ventral midbrain were analyzed using EnrichR. The 2021 Human Gene Ontology library was selected for the analysis; an NHP Gene Ontology library is not currently available. For the temporal lobe, significantly upregulated genes in males when compared to females were analyzed for terms overrepresented in Biological Processes (BP), Molecular Function (MF), Human Phenotype (HP), and Cellular Components (CC). For the cerebellum, significantly downregulated genes in males when compared to females were analyzed for BP, MF, and CC. The top 20 significant GO terms associated with each category (BP, MF, CC, HP) were then inputted into Revigo, a tool that groups similar GO terms together using Multidimensional Scaling to create graphical representations of term similarity. Pathway enrichment analysis for the temporal lobe was generated using EnrichR with the KEGG 2021 Human Pathway library.

## 3. Results

The results reported are for protein-coding genes only. All gene expression analyses were performed as a male expression compared to a female baseline. The temporal lobe was processed and analyzed separately, followed by the ventral midbrain and cerebellum, which were processed together. There is considerable genomic similarity between the species *M. mulatta* (MM) and *M. fascicularis* (CE); sequence reads were run against both transcriptome references to eliminate annotation bias. The CE macaque reference transcriptome left many statistically significant expression hits unannotated compared to the rhesus macaque reference transcriptome, suggesting a potential annotation bias in the CE reference data. Thus, results between the CE and MM transcriptome references were non-redundantly combined to present the most thorough and complete analysis of target genes. For simplification, genes revealed by both references are discussed together with the CE taking priority.

Total RNA-seq analysis on three male and three female macaques, aged between 28 and 32 months, revealed 61 statistically significant (*p* ≤ 0.05) sexually divergent gene expression levels in the temporal lobe. A total of 50/61 genes were overexpressed in males and 11/61 were under expressed; 6 remained unidentifiable after a Basic Local Alignment Search Tool (BLAST) search was performed. Each sample had an average of 99 million raw reads (ranging from 92 to 105 million), with an average mapping rate of 75.43% (ranging from 72.92 to 77.83%). A total of 14,218 protein-coding genes were detected. Table 1 lists all statistically significant differentially expressed genes in males compared to a female baseline within the temporal lobe of the brain. Of note, several interesting genes with functions related to translation were upregulated: Ribosomal Protein S4 Y-linked 1 (RPS4Y1); Ribosomal Protein S4 Y-linked 2 (RPS4Y2); and Eukaryotic Initiation Factor 1A Y-linked (EIF1AY). Genes related to behavior, Arginine Vasopressin (AVP), as well as genes related to immunity—Interleukin 1 Receptor-Associated Kinase 3 (IRAK3), Atypical Chemokine Receptor 1 (ACKR1), Intercellular Adhesion Molecule 1 (ICAM1), MafB Zip transcription factor (MAFB), and Transcription factor AP-1 (JUN)—had statistically significant (*p* < 0.05) differential expression between the sexes.

Pooled samples for the ventral midbrain and cerebellum had an average of 68 million raw reads (ranging from 54 to 85 million for the ventral midbrain and from 51 to 78 million for the cerebellum). The average mapping rate for the cerebellum was 81.82% (ranging from 78.39 to 86.60%). In the cerebellum, total RNA-seq revealed 29 sexually divergent protein-coding genes; 20/29 were upregulated when compared to females and 9/29 were downregulated. Table 2 lists all statistically significant differentially expressed genes in males compared to a female baseline within the ventral midbrain region. Table 3 lists all statistically significant differentially expressed genes in males compared to a female baseline within the cerebellum region. Genes associated with mitochondrial metabolic processes such as Arginosuccinate Synthase 1 (ASS1) and Aldehyde Dehydrogenase 4 family member A1 (ALDH4A1) were downregulated in the male macaques when compared to females.

The average mapping rate for the ventral midbrain was 74.82% (ranging from 73.15 to 77.30%). In the ventral midbrain, total RNA-seq revealed 14 sexually divergent protein-coding genes; 13/14 were upregulated and 1/14 were downregulated.

**Figure 2:**
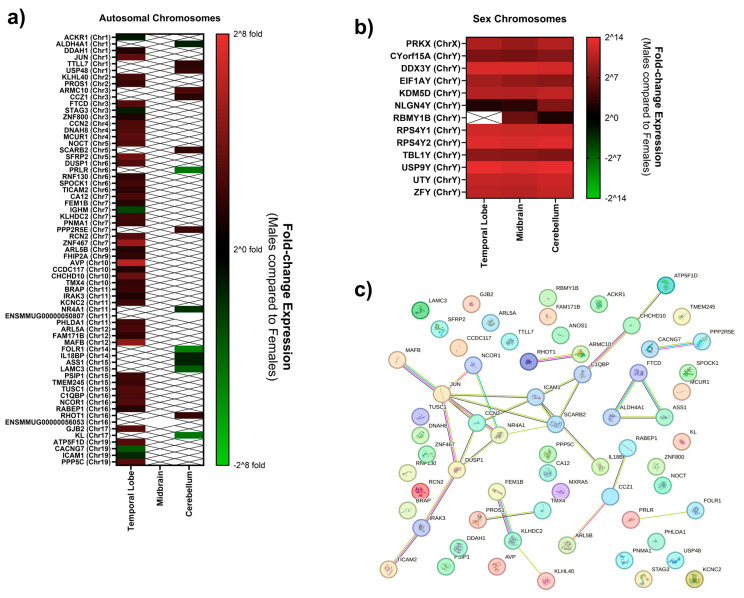
Differential gene expression in males compared to females. Relative gene expression results in statistically significant differentially expressed genes in male macaques compared to female macaques is shown for autosomal chromosomes (**a**) and sex chromosomes (**b**) on a log-base 2 scale across the temporal lobe, midbrain, and cerebellar regions. Genes that were upregulated in males when compared to females range from black to red, with black being mildly upregulated and red being highly upregulated. Genes downregulated in males compared to females range from dark green to light green, with light green being the most downregulated. Respective gene name and chromosome location are denoted to the left of the log scale. Lack of color in a particular brain region indicates that the genes were not significantly differentially expressed there. Genes were cumulatively analyzed using the STRING database to examine the connected pathways. Though genes connected to each other are associated with a particular pathway or process, no statistically significant pathways were identified (**c**).

Cumulative tissue expression for all of the three brain regions analyzed is shown in Figure 2, with genes organized by chromosome of origin. Pathway analysis on differentially expressed genes between males and females was performed for individual brain regions and collectively using the STRING database. Out of the 109 genes affected in total, 99 were annotated in the database when compared against the MM reference, and 51 were annotated when using the CE reference. Two pathways associated with gastrulation and zinc-finger gene regulation reached statistical significance in the cerebellum and ventral midbrain using the MM STRING reference (CL:20229 and CL:20223), while no pathways were identified against the CE STRING reference. These pathway clusters retained statistical significance when analyzing all brain regions simultaneously (*p* < 0.05). While both involve transcription factor regulation of gastrulation, CL:20223 also has strong involvement with oxidative stress, reactive oxygen species, and starvation. To consider translatability to humans, the 109 genes were run against a *Homo sapiens* background. While 95 of these genes were annotated in the STRING database, no statistically significant pathways were enriched.

**Figure 3:**
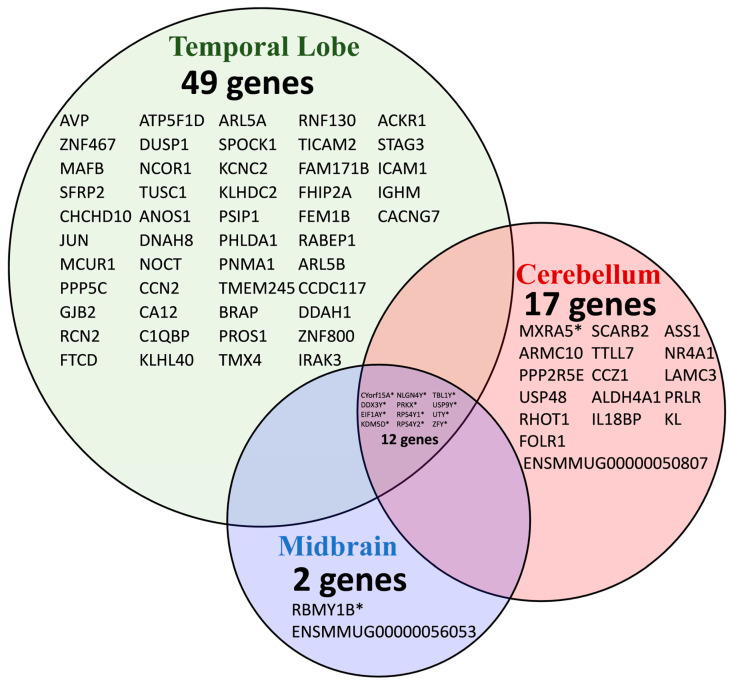
Venn diagram of differentially expressed genes in male and female macaques. The Venn diagram demonstrates the overlap between the statistically significant (*p* < 0.05) sexually divergent transcripts in the temporal lobe, cerebellum, and midbrain. Y-linked genes are annotated with an asterisk (*). Full gene names with respective chromosome functions can be located in Table 1, Table 2 and Table 3.

Figure 3 demonstrates a Venn diagram of the relationship between shared genes in the ventral midbrain, temporal lobe, and cerebellum to illustrate the overlap of sexually divergent, differentially expressed genes between the brain regions investigated. Not surprisingly, all protein-coding genes overlapping amongst the three lobes were Y-linked. Amongst these differentially expressed genes was Neuroligin 4 Y-linked (NLGN4Y), which codes for a cell adhesion protein. Its X-chromosome counterpart has been associated with autism spectrum disorders [20]. The increased expression of NLGN4Y we report in young males—coupled with an absent increased expression of NLGN4X in females—coincides with previous transcriptomic data on sex differences in humans [20,21,22].

**Figure 4:**
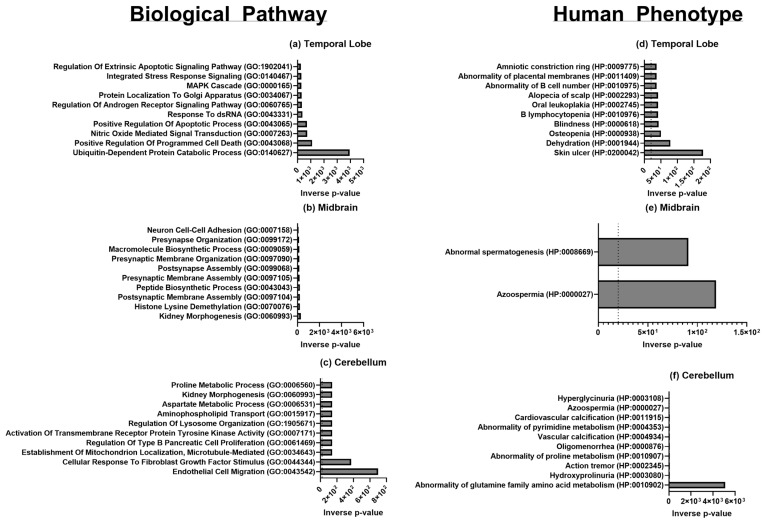
Biological pathway and human phenotype gene ontology by brain location of genes enriched in either male or female macaques using EnrichR and current GO libraries with bar lengths in inverse *p*-value of terms reaching significance for (**a**) temporal lobe, (**b**) midbrain, and (**c**) cerebellum biological pathways and (**d**) temporal lobe, (**e**) midbrain, and (**f**) cerebellum human phenotype ontologic terms.

**Figure 5:**
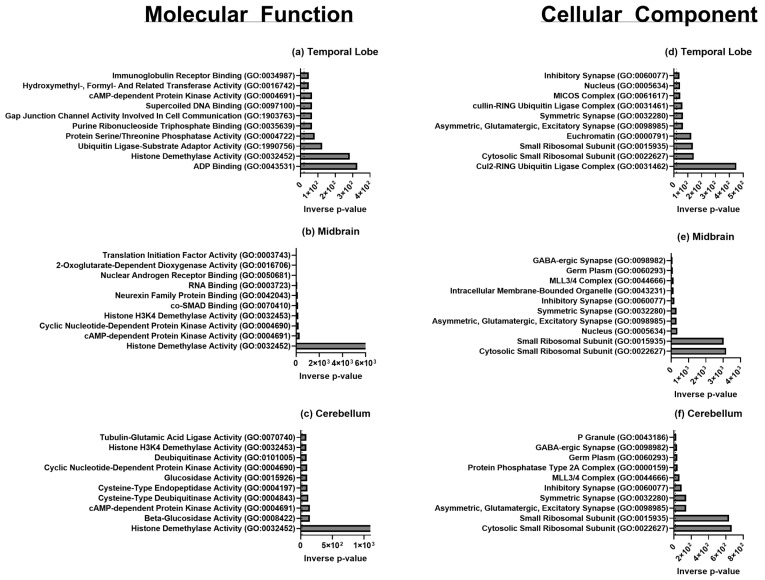
Molecular function and cellular component ontology for sexually divergent transcripts of genes significantly enriched in either male or female macaques using EnrichR and current GO libraries with bar lengths in inverse *p*-value of terms reaching significance for (**a**) temporal lobe, (**b**) midbrain, and (**c**) cerebellum molecular function and (**d**) temporal lobe, (**e**) midbrain, and (**f**) cerebellum cellular-component ontologic terms.

For functional annotation of Gene Ontology (GO), enrichment analysis was performed using EnrichR for terms associated with Biological Processes (BP), Human Phenotype (HP) (Figure 4), Molecular Function (MF), and Cellular Components (CC) (Figure 5). Statistically significant (*p* < 0.05) upregulated and downregulated protein-coding genes in males when compared to females were analyzed to identify overrepresented terms. The below results are based on the GO libraries from the most up-to-date database (2021). GO terms for BP, HP, MF, and CC analyzed by EnrichR [23,24,25] and found to be significantly upregulated from our gene list can be found in Figure 4 and Figure 5. Statistically significant terms generated from EnrichR were then uploaded to Revigo [26,27], where appropriate, to identify and visualize the relationships between GO terms in each category and are also found in Figure 4 and Figure 5. Significant genes from the CE and Rhesus reference transcriptome were combined in the analysis and will be discussed together.

All GO terms and their corresponding GO IDs for all three brain regions are presented in Table 4. Overall, GO terms related to processes of translation and transcription with terms such as demethylase activity and the cytosolic small ribosomal subunit upregulation in males compared to females for all three regions. Additionally, processes related to synaptic functions were consistently upregulated in males in each brain region, though most notably in the temporal region. For the temporal lobe specifically, GO terms relating to the following processes were upregulated in males compared to females: post-translational modifications, immunity, brain development and behavior, and synaptic cellular components. Terms related to MAPK kinase cascade signaling and regulation of programmed cell death, apoptotic process, androgen receptor signaling, and ubiquitination were upregulated.

In the midbrain, GO terms related to the processes of development and cell proliferation were upregulated and citric acid and urea cycles were downregulated in males. Terms related to nucleotide-dependent protein kinase activity, histone methylation, ubiquitination, and synaptic membrane assembly were differentially upregulated in males. Interestingly, the most significantly upregulated terms were related to kidney morphogenesis and azoospermia.

In the cerebellum, processes relating to growth-factor and receptor-binding responses were downregulated in males. Terms of greatest significance related to endothelial cell migration, cellular response to fibroblast growth factor stimulus, and abnormalities of glutamine family amino-acid metabolism.

Statistically significant upregulated and downregulated genes were enriched through EnrichR Biological Pathway Ontology (Figure 4a–c) and Human Phenotype Ontology (Figure 4d–f), with the top 10 associated annotations shown ranked by −log_2_ (*p*-value).

Statistically significant upregulated and downregulated genes were enriched through EnrichR Molecular Function Ontology (Figure 5a–c) and Cellular Component Ontology (Figure 5d–f), with the top 10 associated annotations shown ranked by −log_2_ (*p*-value).

## 4. Discussion

NHPs share many similarities to humans, yet research into the topic of sex differences within brain transcriptomics is not well understood. Genes that confer sex differences in brain development and disease progression could have important implications for human studies. Here, we add to the growing body of knowledge on sexual differences in pre-pubertal CE macaques, using total RNA-seq to evaluate transcriptomic sex differences in the temporal lobe, ventral midbrain, and cerebellum. Analyses of individual genes within each lobe, as well as Gene Set Enrichment Analyses, indicate many protein-coding genes involved in translation, immunity, social behavior, and development are expressed inherently differently between sexes similar to results obtained by others [12]. Our results are distinct from past studies by representation of pre-pubescent macaques and so effects of androgen or estrogen levels on their respective receptor binding site regulated gene transcription are less marked than was observed by studies such as DeCasein et al.; however, similar to these studies, all of the brain regions shared differences in the Y-associated genes [12].

We focused on transcriptomic sex differences, yet significant genes in all three lobes encoded for proteins that are necessary for proper translational function. Eukaryotic Initiation Factor 1A Y-linked (EIF1AY) was highly expressed in all three lobes in male macaques. EIF1AY encodes a protein that helps mediate the efficiency of translation initiation, aiding in both the transfer of a charged met-tRNA to the P site and the formation of the 40 s pre-initiation complex [28]. Previous research in humans comparing the expression of EIF1AY to its X-chromosome homolog, EIF1AX, showed levels were relatively similar amongst tissues, apart from higher expression in the heart, skeletal muscle, spleen, pituitary gland, and blood [29,30]. In the heart, a 5.8-fold increase in expression of EIF1AY was determined to be due to the loss of a target site for a microRNA called miR-1 [24]. While miR-1 is widely expressed in the heart, about 60% of miR-1’s targets are distributed in brain tissue; therefore, a relative deficiency of miR-1 activity to degrade EIF1AY transcripts in the brain could be one explanation for the increased relative expression we report in all three lobes for males compared to females [31].

In addition, Ribosomal S4 Proteins RPS4Y1 and RPS4Y2—which are necessary for the assembly of the 40 s subunit during translation—were upregulated in all three lobes. RPS4Y1 has shown some level of expression in brain vestigial structures [32]. However, previous research on RPS4Y2’s expression in humans limits it to the prostate and testes; this is the first report, to our knowledge, that observes RPS4Y2 expression in the brain of an NHP [29,32]. The expression of genes such as EIF1AY, RPS4Y1, and RPS4Y2 in the brain may point to male differences in translation initiation, elongation efficiency, and regulation; more research is needed to confirm whether heightened expression corresponds to increased translation of these protein-coding genes.

Interesting genes related to immunity also exhibited sexually divergent expression in the temporal lobe. Generally, females tend to have stronger innate and adaptive immune responses than their male counterparts [33]. Thus, we were interested in exploring whether genes in our analysis related to immunity and disease could contribute to our understanding of sex-specific disease development and immunity in an NHP [33]. MAF bZIP Transcription Factor B (MAFB), a gene highly upregulated in the temporal lobe of the male macaques, has been previously reported to play a sex-specific role in severe SARS-CoV-2 infectious disease (COVID-19) [34,35]. Males are generally more susceptible to developing severe COVID-19, yet most discussions on susceptibility focus on co-morbidities as opposed to sex-specific biology to explain this discrepancy [36,37]. MAFB is a basic leucine zipper transcription factor that inhibits the Type 1 Interferon response to viruses. It has been hypothesized that MAFB may excessively suppress this pathway, potentially leading to a critical delay in the immune response during a viral infection. This delay is hypothesized to contribute to severe COVID-19 illness in males [35]. A study on the transcriptomics of COVID-19 patients found MAFB to be significantly upregulated in the serum of patients who were placed in the intensive care unit. Current research shows that even mild COVID-19 infections can cause neurological inflammation [38]. Future research should focus on what role, if any, higher expression levels of MAFB in the brains of males play in this inflammation.

Genes linked to behavior and neurodevelopmental disorders were also identified in the male temporal lobe as sexually divergent; this may be important to our understanding of how neurological disorders develop and present differently amongst the sexes. Of note, the gene Arginine Vasopressin (AVP), a neuropeptide, was upregulated in male CE macaques. AVP has been previously reported to show greater expression in the hypothalamus of rhesus macaques but not in a sexually divergent fashion [28]. Here, we are first to report that sexually divergent AVP expression occurs in the temporal lobe of pre-pubertal male CE macaques. Numerous studies related to AVP’s sex differences in mammals have focused specifically on the hypothalamus, where AVP has been shown to decrease social behavior and communication in males and increase aggression and attempts at establishing dominance [39,40,41,42]. Anecdotally, AVP’s sexually divergent expression may provide insight into behavioral differences observed between males and females. The underlying signaling process for this increased expression and effect on behavior should be explored further in the future. Though the literature on sexual dimorphism in human brain transcriptomics is limited, we sought to compare our results in an NHP to human studies. Trabzuni et al. studied gene expression profiles in the adult human brain and identified the presence of sex differences in 11 brain regions [22]. When comparing significant genes in Trabzuni’s study to our CE macaques, nine genes were shared: DDX3Y, EIF1AY, KDM5D, NLGN4Y, RPS4Y1, USP9Y, UTY, and ZFY. EIF1AY and RPS4Y1 were previously discussed in the context of translation but this shared finding to a human study adds a degree of translatability. Further, the authors note that NLGN4Y was overexpressed in human males and CE macaques, and has X-linked counterparts, although NLGN4X should show overexpression in females if the gene expression profiles amongst males and females are balanced for these genes; however, it was not overexpressed in females in the human nor macaque study, providing further evidence of unequal dosing of this gene pair across species.

This study was conducted alongside an AAV vector transduction comparison in support of animal use reduction. Animals were injected intracranially with a barcoded mix of AAV vectors; intracranial injection and/or AAV treatment may have influenced gene expression. However, we propose the gene expression differences we observed in the brain are primarily due to innate sex differences and not purely a response to the treatment of AAV since all animals received the same AAV injection mix and the proportion of RNA transcripts in these regions from the AAV luciferase/mApple reporter transgenes was less than 0.001%. To our knowledge, research into gene expression changes in the brain following the treatment of AAV vectors does not currently exist. It is possible that the expected immune response from the intracranial AAV injection could have exacerbated expression differences between males and females, especially in protein-coding genes that function to activate the immune system. However, it is worth noting that AAV at the doses injected does not typically generate strong immune reactions when compared to other gene therapy options such as adenoviral vectors.

The macaques in this study are pre-pubertal, therefore, the effects of age on gene expression cannot be ruled out. This study was also limited to three brain regions and had a focus on protein-coding genes; transcriptomic profiles in more detailed structures of the brain would provide additional region-specific information on gene expression, such as has been conducted in adult animals [12]. Further, bulk RNAseq does not provide information of transcriptional differences between individual cells, and thus does not account for differences in neuronal density between males and females that may explain the upregulation of terms related to synaptic function in all brain regions tested. Nor does bulk RNAseq detect small non-protein-coding RNA such as miRNA and siRNA; these ncRNAs are important to transcriptional regulation such as splicing and silencing of mRNA. Most ncRNAs can be detected through single-cell RNAseq but this analysis was not feasible due to cost constraints for this study. However, understanding transcriptomic changes within individual cells and cell types within individual brain regions would greatly improve this study. We propose a future spatio-temporal study that compares age, regions, and sex with total and single-cell RNAseq; this would add to our understanding of gene expression, regulation, and brain development in an NHP.

### Perspectives and Significance

Given the similarity in the transcriptomic profile of humans and macaques, the results of this study can be applied broadly to future research studies on CNS transcriptomics in humans and non-human primates. It may be a baseline to rule out genes that could pose sex-specific confounds to a study or inform researchers of sexual differences in their genes of interest. Most notably, mechanisms for sex differences in disease development, progression, and outcomes can be explored based on the target genes we identify here. The treatment of diseases with sex-specific etiology cannot be fully understood without an understanding of sexually divergent expression differences in health.

## 5. Conclusions

Research using CE macaques continues to be a valuable model for human physiology and disease. Overall, this study adds to the growing body of literature on the presence of sexual differences in the brains of pre-pubertal non-human primates. Differences in genes associated with immunity, social behavior, aggression, and dominance were observed. Results of gene ontology show that GO terms related to translation, immune response to disease, and synaptic function were differentially expressed in males compared to females. Translational upregulation in males may be, in part, due to the fact that males have slightly larger brains than women; thus, more proteins are likely to be translated. Differences in immune response to disease are concurrent with individual gene functions highlighted previously in the Discussion Section. Future research is necessary to confirm these findings and should focus on how sexually divergent expression profiles contribute to differences in the presentation of diseases in humans.

## Figures and Tables

**Table 1:** Differentially expressed protein-encoding genes in the temporal lobe of males compared to females.

Gene Symbol	Gene Name	Chromosome Location	Log_2_ (FC)	−Log (*p*-Value)
USP9Y	ubiquitin-specific peptidase 9, Y-linked	Y	13.71905899	6.50266 × 10^25^
RPS4Y2	Ribosomal Protein S4, Y-linked 2	Y	13.10653319	9.8653 × 10^23^
DDX3Y	DEAD-box helicase 3 Y-linked	Y	12.64835585	4.51949 × 10^21^
RPS4Y1	Ribosomal Protein S4 Y-linked	Y	12.11459869	2.51384 × 10^19^
UTY	Ubiquitously transcribed tetratricopeptide repeat containing, Y-linked	Y	11.3635802	7.46989 × 10^17^
ZFY	Zinc Finger Protein Y-linked	Y	10.92233302	1.05005 × 10^14^
KDM5D	lysine demethylase 5D	Y	10.46017162	8.09787 × 10^14^
PRKX	cAMP-dependent protein kinase catalytic subunit PRKX	X	10.02681145	3.47977 × 10^12^
EIF1AY	Eukaryotic Initiation Factor 1AY	Y	9.560043266	4.51949 × 10^21^
TBL1Y	transducin beta-like 1 Y-linked	Y	8.251471122	3.63009 × 10^7^
CYorf15A	Chromosome Y Open Reading Frame 15A	Y	7.266208729	0.00042528
AVP	Vasopressin-neurophysin 2-copeptin	10	6.303191895	0.029333155
ZNF467	Zinc Finger Protein 467	7	5.246107131	0.015446581
MAFB	MAF bZIP transcription factor B	12	4.754125701	0.00011165
SFRP2	Secreted frizzled-related protein 2	5	3.834735022	0.004961535
CHCHD10	Coiled-coil-helix-coiled-coil-helix domain-containing protein 10, mitochondrial	10	3.536977542	0.003289907
JUN	Transcription factor AP-1	1	3.297279849	0.001233038
MCUR1	mitochondrial calcium uniporter regulator 1	4	3.062017972	0.011472793
PPP5C	Serine/threonine-protein phosphatase 5	19	3.025384368	4.55417 × 10^5^
GJB2	Gap junction beta-2 protein	17	2.959247277	2.38261 × 10^5^
RCN2	reticulocalbin 2	7	2.917966017	0.028275537
FTCD	formimidoyltransferase cyclodeaminase	3	2.909467024	0.048848313
ATP5F1D	ATP synthase subunit delta, mitochondrial	19	2.908590716	0.037687724
DUSP1	Dual specificity protein phosphatase 1	6	2.844496222	0.002164976
NCOR1	Nuclear receptor corepressor 1	16	2.819544283	0.003434749
TUSC1	tumor suppressor candidate 1	15	2.805361398	0.02291493
ANOS1	Anosmin 1	X	2.757610957	0.013726134
DNAH8	Dynein axonemal heavy chain 8	4	2.742246473	9.42881 × 10^5^
NOCT	Nocturnin	5	2.733568645	0.005494721
CCN2	cellular communication network factor 2	4	2.652254082	0.010661883
CA12	Carbonic anhydrase 12	7	2.64452723	0.008978305
C1QBP	Complement C1Q Binding Protein	16	2.499674658	0.018091751
KLHL40	Kelch-like protein 40	2	2.490703418	0.032617475
ARL5A	ADP-ribosylation factor-like protein 5A	12	2.481455264	0.010661883
NLGN4Y	Neuroligin-4, Y-linked	Y	2.303917734	3.43641 × 10^9^
SPOCK1	SPARC (osteonectin), cwcv- and kazal-like domains proteoglycan 1	6	2.296600371	0.037687724
KCNC2	Potassium voltage-gated channel subfamily C member 2	11	2.25107452	0.048848313
KLHDC2	Kelch domain-containing protein 2	7	2.231606579	0.002213669
PSIP1	PC4 and SFRS1 interacting protein 1	15	2.183075899	0.042711587
PHLDA1	Pleckstrin homology-like domain family A	11	2.151384308	0.029512147
PNMA1	Paraneoplastic antigen Ma1	7	2.145699785	0.017094243
TMEM245	Transmembrane protein 245	15	2.07936194	0.001884686
BRAP	BRCA1-associated protein	11	2.038348427	0.040753462
PROS1	Protein S	2	1.923748541	0.017094243
TMX4	Thioredoxin-related transmembrane protein 4	10	1.822929354	0.003130618
RNF130	E3 ubiquitin-protein ligase RNF130	6	1.724726953	0.042631739
TICAM2	TIR domain-containing adapter molecule 2	6	1.687826371	0.015446581
FAM171B	Protein FAM171B	12	1.684187452	0.015203882
FHIP2A	FHF complex subunit HOOK interacting protein 2A	9	1.655411608	0.047315236
FEM1B	fem-1 homolog B	7	1.563853061	0.049277464
RABEP1	rabaptin, RAB GTPase binding effector protein 1	16	1.469789211	0.02291493
ARL5B	ADP ribosylation factor like GTPase 5B	9	1.442835309	0.048848313
CCDC117	Coiled-coil domain-containing protein 117	10	1.395598027	0.015488608
DDAH1	N(G),N(G)-dimethylarginine dimethylaminohydrolase 1	1	1.304359376	0.013726134
ZNF800	Zinc Finger Protein 800	3	1.264760526	0.010661883
IRAK3	Interleukin 1 Receptor-Associated Kinase 3	11	1.137212821	0.015446581
ACKR1	Atypical Chemokine Receptor 1	1	−1.285715044	0.042631739
STAG3	STAG3 cohesin complex component	3	−1.33655274	0.047315236
ICAM1	Intercellular Adhesion Molecule 1	19	−1.765898547	0.028275537
IGHM	Immunoglobulin heavy constant mu	7	−2.870128518	0.007092621
CACNG7	calcium voltage-gated channel auxiliary subunit gamma 7	19	−3.423844655	0.019990396

**Table 2:** Differentially expressed protein-encoding genes in the ventral midbrain of males compared to females.

Gene Symbol	Gene Name	Chromosome Location	Log_2_ (FC)	−Log (*p*-Value)
USP9Y	ubiquitin-specific peptidase 9 Y-linked	Y	13.10632061	7.79953 × 10^23^
DDX3Y	ubiquitin-specific peptidase 9 Y-linked	Y	12.64865658	3.39235 × 10^21^
RPS4Y2	Ribosomal Protein S4, Y-linked 2	Y	12.61650867	1.31892 × 10^20^
RPS4Y1	Ribosomal Protein S4, Y-linked 1	Y	11.86763536	1.58552 × 10^17^
UTY	Ubiquitously transcribed tetratricopeptide repeat containing, Y-linked	Y	11.26290341	2.01041 × 10^16^
ZFY	Zinc Finger Protein Y-linked	Y	10.64300771	1.04026 × 10^14^
KDM5D	lysine demethlyase 5D	Y	10.40639548	9.19827 × 10^14^
PRKX	cAMP-dependent protein kinase catalytic subunit PRKX	X	8.806541644	1.18975 × 10^8^
EIF1AY	Eukaryotic Initiation Factor 1A Y-linked	Y	8.580239692	6.16063 × 10^29^
TBL1Y	transducin beta-like 1 Y-linked	Y	7.939914094	2.44015 × 10^6^
CYorf15A	Chromosome Y Open Reading Frame 15A	Y	6.946916081	0.000634637
RBMY1B	RNA-binding motif protein, Y chromosome, family 1 member B-like	Y	6.253240916	0.015123427
NLGN4Y	Neuroligin-4, Y-linked	Y	2.818620993	1.58552 × 10^17^
ENSMMUG00000056053	Unknown	16	−8.061325309	0.00587965

**Table 3:** Differentially expressed protein-encoding genes in the cerebellum of males compared to females.

Gene Symbol	Gene Name	Chromosome Location	Log_2_ (FC)	−Log (*p*-Value)
USP9Y	ubiquitin-specific peptidase 9 Y-linked	Y	13.53358026	1.69166 × 10^24^
RPS4Y2	Ribosomal Protein S4, Y-linked 2	Y	13.05561903	4.36744 × 10^22^
DDX3Y	ubiquitin-specific peptidase 9 Y-linked	Y	12.24246642	2.65269 × 10^21^
RPS4Y1	Ribosomal Protein S4, Y-linked 1	Y	11.98614904	6.36374 × 10^18^
UTY	Ubiquitously transcribed tetratricopeptide repeat containing, Y-linked	Y	11.90810859	1.99374 × 10^18^
ZFY	Zinc Finger Protein Y-linked	Y	11.53537061	1.54245 × 10^17^
KDM5D	lysine demethlyase 5D	Y	11.21577309	3.18329 × 10^16^
PRKX	cAMP-dependent protein kinase catalytic subunit PRKX	X	10.42609013	8.28047 × 10^13^
EIF1AY	Eukaryotic Initiation Factor 1A Y-linked	Y	7.937725996	2.13809 × 10^32^
CYorf15A	Chromosome Y Open Reading Frame 15A	Y	7.648642346	3.52159 × 10^5^
MXRA5	Matrix Remodeling-Associated Protein 5	Y	7.535908795	1.73413 × 10^7^
TBL1Y	transducin beta-like 1 Y-linked	Y	7.463601541	1.86266 × 10^5^
ARMC10	armadillo repeat containing 10	3	2.622782332	0.015667643
PPP2R5E	protein phosphatase 2 regulatory subunit B’epsilon	7	2.132210121	0.04670821
NLGN4Y	neuroligin 4, Y-linked	Y	1.951104573	5.15756 × 10^8^
USP48	ubiquitin-specific peptidase 48	1	1.889296185	0.027143557
RHOT1	ras homolog family member	16	1.85772343	0.035628187
SCARB2	scavenger receptor class B member 2	5	1.798621645	0.021182753
TTLL7	tubulin tyrosine ligase like	1	1.614680379	0.028528043
CCZ1	Vacuolar Protein Trafficking and Biogenesis Associated	3	1.508827318	0.020455655
ALDH4A1	Aldehyde Dehydrogenase 4 family member A1	1	−1.438885962	0.03303172
IL18BP	interleukin 18 binding protein	14	−1.507517697	0.014251736
ASS1	Argininosuccinate synthase	15	−1.629165355	0.034521802
NR4A1	Nuclear receptor subfamily 4 group A member 1	11	−2.100052055	0.00555242
LAMC3	Laminin Subunit Gamma 3	15	−3.588868028	2.58109 × 10^8^
PRLR	prolactin receptor	6	−4.548855035	0.03946567
KL	Klotho	17	−4.572208799	0.00555242
FOLR1	Folate receptor alpha	14	−4.999557377	0.009471893
ENSMMUG00000050807	Unknown	11	−22.10508155	0.000187717

**Table 4:** GO terms upregulated or downregulated across different brain regions and associated processes.

Brain Region	GO Term and ID	Upregulated/Downregulated	Associated Process
Temporal Lobe, Cerebellum, Midbrain	translation (GO: 0006412)	Upregulated	Translation
Temporal Lobe, Midbrain	peptide biosynthetic process (GO: 0043043)	Upregulated	Translation
Temporal Lobe	SRP-dependent co-translational protein targeting to the membrane (GO: 0006614)	Upregulated	Translation
Temporal Lobe	large ribosomal subunit (GO: 0015934)	Upregulated	Translation
Temporal Lobe, Cerebellum, Midbrain	small ribosomal subunit (GO: 0015935)	Upregulated	Translation
Temporal Lobe, Midbrain	RNA binding (GO: 0003723)	Upregulated	Transcription
Temporal Lobe	histone lysine demethylation (GO: 0070076)	Upregulated	Transcription
Temporal Lobe	histone H3-K27 demethylation (GO: 0071557)	Upregulated	Transcription
Temporal Lobe	gene expression (GO: 0010467)	Upregulated	Transcription
Temporal Lobe	phosphatase activity (GO: 0016791)	Upregulated	Post-translational Modifications
Temporal Lobe	peptidyl-serine dephosphorylation (GO: 0070262)	Upregulated	Post-translational Modifications
Temporal Lobe	peptidyl-threonine dephosphorylation (GO: 0035970)	Upregulated	Post-translational Modifications
Temporal Lobe	CAMP-dependent protein kinase activity (GO: 0004691)	Upregulated	Post-translational Modifications
Temporal Lobe	ubiquitin protein ligase activity (GO: 0061630)	Upregulated	Post-translational Modifications
Temporal Lobe	regulation of complement activation (GO: 0030449)	Upregulated	Immunity
Temporal Lobe	regulation of immune effector process (GO: 0002697)	Upregulated	Immunity
Temporal Lobe	positive regulation of leukocyte chemotaxis (GO: 0002690)	Upregulated	Immunity
Temporal Lobe	regulation of humoral immune response (GO: 0002920)	Upregulated	Immunity
Temporal Lobe	regulation of androgen receptor signaling pathway (GO: 0060765)	Upregulated	Brain Development and Behavior
Temporal Lobe	dopamine neurotransmitter receptor activity (GO: 0004952)	Upregulated	Brain Development and Behavior
Temporal Lobe	asymmetric synapse (GO: 0032279)	Upregulated	Synaptic Cellular Components
Temporal Lobe	asymmetric glutamatergic, excitatory synapse (GO: 0098985)	Upregulated	Synaptic Cellular Components
Temporal Lobe	symmetric synapse (GO: 0032280)	Upregulated	Synaptic Cellular Components
Temporal Lobe	inhibitory synapse (GO: 0060077)	Upregulated	Synaptic Cellular Components
Cerebellum, Midbrain	cytosolic small ribosomal subunit (GO: 0022627)	Upregulated	Translation
Cerebellum	histone demethylase activity (GO: 0032452)	Upregulated	Translation
Cerebellum	debiquitinase activity (GO: 0101005)	Upregulated	Translation
Cerebellum	cellular response to fibroblast growth factor stimulus (GO: 0044344)	Downregulated	Growth Factors and Receptor Binding
Cerebellum	glucocorticoid receptor binding (GO: 0035259)	Downregulated	Growth Factors and Receptor Binding
Cerebellum	fibroblast growth factor binding (GO: 0017134)	Downregulated	Growth Factors and Receptor Binding
Cerebellum	growth factor receptor binding (GO: 0070851)	Downregulated	Growth Factors and Receptor Binding
Cerebellum	nuclear receptor binding (GO: 0016922)	Downregulated	Growth Factors and Receptor Binding
Midbrain	aspartate metabolic process (GO: 0006531)	Downregulated	Citric Acid and Urea Cycle
Midbrain	tricarboxylic acid metabolic process (GO: 0072350)	Downregulated	Citric Acid and Urea Cycle
Midbrain	arginine metabolic process (GO: 0006525)	Downregulated	Citric Acid and Urea Cycle
Midbrain	citrulline metabolic process (GO: 0000052)	Downregulated	Citric Acid and Urea Cycle
Midbrain	urea cycle (GO: 0000050)	Downregulated	Citric Acid and Urea Cycle
Midbrain	kidney morphogenesis (GO: 0060993)	Upregulated	Development and Cell Proliferation
Midbrain	determination of pancreatic left/right asymmetry (GO: 0035469)	Upregulated	Development and Cell Proliferation
Midbrain	determination of liver left/right asymmetry (GO: 0071910)	Upregulated	Development and Cell Proliferation
Midbrain	determination of digestive tract left/right symmetry (GO: 0071907)	Upregulated	Development and Cell Proliferation
Midbrain	ureter development (GO: 0072189)	Upregulated	Development and Cell Proliferation
Midbrain	cardiac atrium development (GO: 0003230)	Upregulated	Development and Cell Proliferation
Midbrain	nuclear androgen receptor binding (GO: 0050681)	Upregulated	Development and Cell Proliferation
Midbrain	regulation of Wnt, planar cell polarity pathway (GO: 2000095)	Upregulated	Development and Cell Proliferation

## Data Availability

All data generated or analyzed during this study are included in this published article.

## Abbreviations

The following abbreviations are used in this manuscript:

RNA	Ribonucleic acid
AAV	Adeno-associated virus
NHP	Non-human primate
MRI	Magnetic Resonance Imaging
MM	Rhesus macaques or *M. mulatta*
CE	Cynomolgus macaques or *M. fascicularis*
IACUC	Institutional Animal Care and Use Committee
DNA	Deoxyribonucleic acid
ICBR	Interdisciplinary Center for Biotechnology Research
NGS	NextGen DNA Sequencing
BP	Biological Processes
MF	Molecular Function
HP	Human Phenotype
CC	Cellular Components
GO	Gene Ontology
BLAST	Basic Local Alignment Search Tool
RPS4Y1	Ribosomal Protein S4 Y-linked 1
RPS4Y2	Ribosomal Protein S4 Y-linked 2
EIF1AY	Eukaryotic Initiation Factor 1A Y-linked
AVP	Arginine Vasopressin
IRAK3	Interleukin 1 Receptor-Associated Kinase 3
ACKR1	Atypical Chemokine Receptor 1
ICAM1	Intercellular Adhesion Molecule 1
MAFB	MafB Zip transcription factor
JUN	Transcription factor AP-1
ASS1	Arginosuccinate Synthase 1
ALDH4A	1 Aldehyde Dehydrogenase 4 family member A1
NLGN4Y	Neuroligin 4 Y-linked
NLGN4X	Neuroligin 4 X-linked
NIH	National Institutes of Health
NINDS	National Institutes of Neurological Disorders and Stroke
